# Prevalence of *Salmonella* spp., *Shigella* spp., and intestinal parasites among food handlers working in University of Gondar student’s cafeteria, Northwest Ethiopia

**DOI:** 10.3389/fpubh.2024.1370338

**Published:** 2024-05-01

**Authors:** Azanaw Amare, Setegn Eshetie, Desie Kasew, Ayenew Amare, Wondwossen Abebe, Feleke Moges

**Affiliations:** ^1^Department of Medical Microbiology, School of Biomedical and Laboratory Sciences, College of Medicine and Health Sciences, University of Gondar, Gondar, Ethiopia; ^2^Department of Laboratory Sciences, Bahir Dar Health Sciences College, Bahir Dar, Ethiopia

**Keywords:** *Salmonella* species, *Shigella* species, intestinal parasites, multi-drug resistant, food handler, Gondar

## Abstract

**Background:**

Food-borne infections continue to be a major public health problem at the international level. The issue becomes more serious in developing countries like Ethiopia.

**Objective:**

This study aimed to examine the prevalence of *Salmonella* and *Shigella* species and intestinal parasites, as well as antimicrobial resistance patterns and associated factors among food handlers at the University of Gondar cafeteria in northwest Ethiopia.

**Methods:**

An institutional-based cross-sectional study was conducted from February to June 2021 in the University of Gondar cafeterias. Data related to the socio-demographic characteristics and hygienic practices of study participants were collected using structured questionnaires. A total of 290 stool samples were collected from food handlers. Culture and conventional biochemical tests were used to isolate the *Salmonella* and the *Shigella* species. Wet mount, Formol-ether concentration, and Kato Katz techniques were applied to identify intestinal parasites. Additionally, drug susceptibility tests were performed using the disk diffusion method. Statistical analysis was done using SPSS version 26.

**Results:**

Of 290 food handlers’ stool samples analyzed, Twenty-seven 27 (9.3%) were positive for both *Salmonella* and *Shigella* species. The prevalence of *Salmonella* and *Shigella* species was 16 (5.5%) and 11 (3.8%), respectively. Most of the isolated pathogens were resistant to tetracycline 19 (70.4%), and trimethoprim/sulphamethoxazole 19 (70.4%). The overall rate of multi-drug resistant *Shigella* and *Salmonella* isolate was 59.3%. Besides, Fifty-seven 57 (19.7%) of the participants were positive for one or more intestinal parasites. The most prevalent intestinal Parasitosis was *E. histolytica/dispar* 22 (7.6%), followed by *G. lamblia* 13 (4.5%), and *Ascaris lumbricoides* 11 (3.8) not washing hands after using the toilet (AOR: 4.42, 95% CI: 1.57, 10.56), and consuming unpasteurized milk (AOR: 3.14, 95% CI: 1.65, 3.96), were factors significantly associated with the prevalence of Salmonella, and Shigella infection. Similarly, not washing hands after using the toilet (AOR: 2.19, 95% CI: 1.0, 1.4), and consuming unpasteurized milk (AOR: 10.4, 95% CI: 3.8, 28.8), were factors significantly associated with the prevalence of intestinal parasites infection.

**Conclusion:**

The prevalence of intestinal parasites, *Salmonella*, and *Shigella* species was high. Therefore, it is imperative to implement a public health policy that includes ongoing microbiological surveillance.

## Introduction

Food-borne diseases (FBD) refer to illnesses caused by consuming pathogenic microorganisms, such as bacteria, fungus, viruses, and parasites, or their toxins in the case of bacteria and fungi (1–3). Additionally, FBD is caused by unprotected food handling and processing, and poor Sanitary conditions (4). It has recently become a worldwide and local health problem. Every year, two million deaths from food-borne illnesses are reported, affecting around one-third of the world’s population (5). Across both developed and developing countries, it is a major public health concern (5). The World Health Organization (WHO) states that 600 million people worldwide become severely ill each year, with 420,000 dying as a result of food contamination. FBD affects an estimated 48 million people in the United States each year, resulting in 128,000 hospitalizations and 3,000 fatalities (6, 7). Food-borne and waterborne infections induce diseases that are both short-term (such as nausea, vomiting, and diarrhea) and long-term (such as cancer, kidney or liver failure, tissue damage, brain disorders, and neurological abnormalities) and are also projected to cause approximately 700,000 deaths each year in Africa (8).

Gastrointestinal parasitic infections are prevalent in both developed and developing countries, with the largest frequency in less developed countries as a result of socioeconomic, demographic, and health-related behaviors, as well as inadequate personal hygiene and environmental sanitation (9). The most common way for intestinal parasitic infections to spread is through contaminated food and water, but they can be further transmitted from person to person through feco-oral contact (10). Nearly one-third of the populations in affluent countries suffer intestinal infections caused by parasites (11). *Ascaris lumbricoides*, *Trichuris trichiura*, hookworm, *Entamoeba histolytica*, and *Giardia lamblia* infect an estimated 1.2 billion, 795 million, 740 million, 500 million, and 2.8 million people worldwide, respectively (12, 13). In Ethiopia, the burden of intestinal parasites (IPs) is extremely high. A third (26 million), a quarter (21 million), and one in every eight (11 million) Ethiopians are infected with *Ascaris lumbricoides*, *Trichuris trichiura*, and hookworm, respectively (14–16). As a result, Ethiopia has the second, third, and fourth largest burdens of ascariasis, hookworm, and trichuriasis, in sub-Saharan Africa, respectively (17).

Food-borne disease is also caused by enteric bacterial infections (EBIs), including those caused by the genus Shigella (which causes Shigellosis) and Salmonella (which causes Salmonellosis). They thus remain important public health concerns. Moreover, vaccinations do not produce immunity in young infants, and the development of antibiotic resistance makes clinical prevention and control of typhoid fever challenging, especially in Africa (18). Every year, *Salmonella* species cause 93.8 million episodes of gastroenteritis worldwide, resulting in 155,000 fatalities. Of these instances, 80.3 million were thought to be food-borne (19). *Shigella* species are more common in temperate and tropical areas. *Shigella* species cause an estimated 80–165 million cases of disease and 600,000 deaths worldwide each year (20). *Salmonella* and *Shigella s*pecies resistant to commonly prescribed antibiotics are a threat to children and the general community (21). Ethiopia has been reported to exhibit considerable rates of resistance for *Shigella* and *Salmonella* species to tetracycline (52.5, 82.4%), trimethoprim/sulphamethoxazole (37.5, 76.5%), and ampicillin (60, 47.1%), respectively (22).

Food handlers are those who work in the food preparation and serving industry. If they have bacterial or parasite diseases in their gastrointestinal tract and maintain poor personal hygiene, they may pose a major risk of spreading IPs and EBIs to the general public. Asymptomatic food handlers are known to play an essential role in the spread of infections and continue to pose a hazard to the public’s health (23–26). Previous research has shown that food prepared in large quantities by a variety of food handlers at higher education institutions is frequently prone to contamination by infectious microorganisms or asymptomatic carriers, which can cause outbreaks of food-borne diseases (24, 25). Furthermore, due to insufficient laboratory infrastructure that impedes effective detection and antibiotic susceptibility testing, *Shigella*, and *Salmonella* species isolation in most African laboratories, including Ethiopia, continues to be difficult (27). As a result, there is a dearth of current information on *Shigella*, *and Salmonella* species, and their patterns of antibiotic susceptibility, as well as the prevalence of intestinal parasites among Ethiopian food handlers. Therefore, the purpose of this study was to evaluate the frequency of intestinal parasites, *Shigella*, and *Salmonella* species in the University of Gondar cafeteria, as well as the patterns of antimicrobial resistance of the isolates along with associated factors.

## Methods and materials

### Study setting and period

An institutional-based cross-sectional study was carried out among food handlers working at the University of Gondar Hospital and Student Cafeteria, northwest, Ethiopia from February to June 2021. The University of Gondar is located in Gondar at a distance of 175 from Bahir Dar and 740 km from Addis Ababa. Currently, the University of Gondar Hospital and Student Cafeteria serve meals to over 37,000 students and staff.

### Study population, sample size, and sampling technique

The source populations were all food handlers at the University of Gondar Hospital and the student Cafeteria, handled food. The study populations were food handlers who worked in the cafeteria at the University of Gondar, were available at the time of data collection, and gave their consent. Food handlers who used antibiotics and/or anthelminthic drugs and were not have willingness to give stool samples were also excluded during sample collection.

The sample size was determined by using a single population proportion formula considering the following assumptions: Zα/2 = 1.96 for the standard scale of 95% level of confidence, level of precision (d) = 3%, p = expected prevalence don by Mama and Alemu (6.9%) (28).


$$n=\frac{\left(Z\alpha l2\right)2p\left(1-p\right)}{\left(d\right)2}\;=\;\frac{\left(1.96\right)2*0.069\left(0.931\right)}{\left(0.03\right)2}=274.2\text{.}$$

With a 5% non-response rate = 290. As a result, 290 food handlers from all campus cafeterias were included in the study. A simple random sampling method was used to select the study participants. A stratified random sampling technique was used to recruit 290 study participants from the sampling frame of food handlers The number of food handlers chosen from each cafeteria was as follows: Hospital (*n* = 120), College of Medicine and Health Science campus (*n* = 108), Atse tewodros campus (*n* = 138), Maraki campus (*n* = 186), Fasile campus (*n* = 52), and Tseda campus (*n* = 45). To select representative participants, the final sample size was proportionally allocated to each stratum using the formula donated below. $ni=\frac{n}{N}xNi\text{,}$ Where n = total sample size to be selected, N = total population, Ni = total population of each stratum, and ni = sample size from each strata. Then the final sample size chosen from each cafeteria from the hospital, College of Medicine and Health Science campus, Atse Tewodros campus, Maraki campus, Fasile campus, and Tseda campus were 54, 48, 83, 62, 23, and 20 food handlers, respectively.

### Data collection methods and tools

#### Socio-demographic data collection

Using a standardized questionnaire, data regarding socio-demographic characteristics and associated factors such as gender, age, education level, marital status, service years, medical checkups, the practice of hand washing after the toilet, hand washing using soap, fingernail status, wearing hair cover, the practice of drinking unpasteurized milk, eating raw meat, and eating raw vegetables were collected. The questionnaires’ validity was checked for accuracy and completeness by conducting a pretest at Wogera prison center.

#### Specimen collection, handling, and transportation

Using a labeled, clean, wide-mouth plastic container and a clean, wooden applicator stick, 3 g of fresh stool samples were collected from food handlers per the recommended procedures (28). Then the samples were subsequently preserved in Cary Blair media (for culture) and a 10% (V/V) formalin solution (for wet mount and parasite concentration) was used. The samples were then promptly transported to the laboratory using a sample carrier.

#### Specimen processing and examination

##### Parasite identification

To identify intestinal protozoa, each sample was immediately analyzed with normal saline (0.85% NaCl) and Lugol’s iodine. A Formol-ether concentration method and Kato Katz methods were employed to enhance the detection of intestinal parasites (28).

##### Isolation and identification of *Shigella* and *Salmonella* species

**Figure 1:**
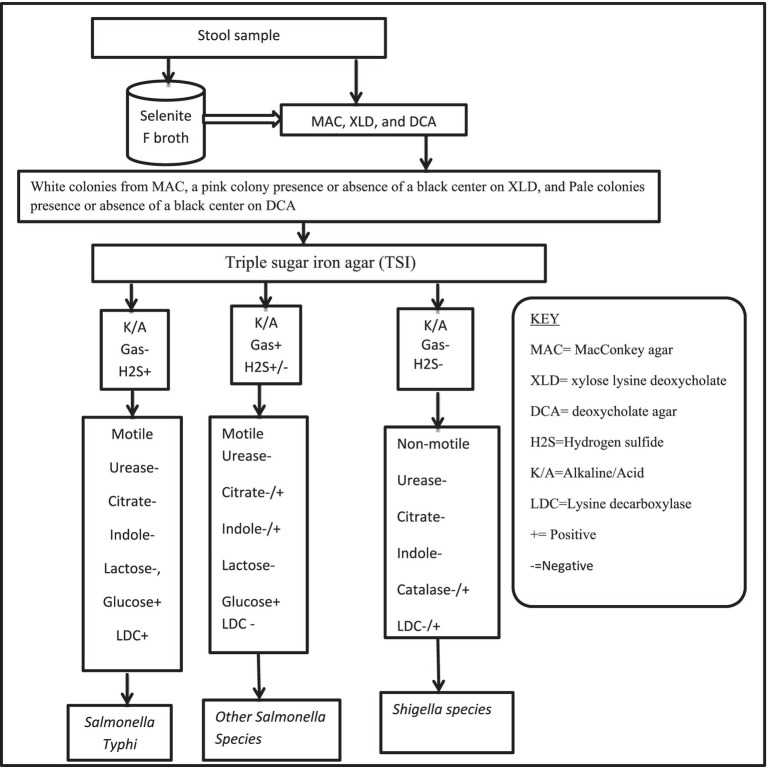
Flow chart used for identification of *Salmonella* and *Shigella* species.

Using the standardized technique, *Salmonella*, and *Shigella* species were characterized and isolated (29). To enrich the bacteria, one milliliter of a stool sample was mixed with nine milliliters of Selenite F broth after being transferred from the Cary Blair medium (Oxoid, Ltd. UK) and incubated at 37°C for 24 h. MacConkey agar (21) (Oxoid, Ltd., UK), deoxycholate agar (DCA), and xylose lysine deoxycholate (XLD) agar (Oxoid, Ltd., UK) were used to subculture an inoculum from selenite F broth. After 24 h of incubation at 37°C, the growth of *Salmonella* and *Shigella* species was differentiated by their colony characteristic appearance on DCA agar (*Shigella* species shows pale colonies in color, *Salmonella* species black center pale color colonies) and XLD agar (Shigella has red color colonies, *Salmonella* species red with a presence of black color center). Discreet pure colonies with a characteristic of *Salmonella*- and/or *Shigella*-like species were further inoculated into standard biochemical tests such as Triple Sugar Iron Agar (TSI) (Oxoid, Ltd., UK), Sulfide Indole Motility Agar (SIM) (Oxoid, Ltd., UK), Urea Agar (Oxoid, Ltd., UK), Simmons Citrate Agar (Oxoid, Ltd., UK), and Lysine iron Agar (Oxoid, Ltd., UK) for species identification. Subsequently, according to the established technique, the results of the biochemical test results consistent with *Salmonella* and/or *Shigella* species were reported (Figure 1) (30, 31). Then discreet pure colonies presence or absence of black centered on DCA or XLD were picked and suspended in sterile normal saline (0.85% NaCl) for an antimicrobial susceptibility test (AST).

### Antimicrobial susceptibility testing

Once the bacteria had been identified, the isolates’ ASTs were carried out using a modified Kirby-Bauer disk diffusion technique following the recommendation of the Clinical and Laboratory Standard Institute (CLSI), 2020. The bacterial suspension was prepared by emulsifying pure colonies from a young culture in 0.85% sterile normal saline. This suspension was then compared to 0.5 McFarland turbidity standards (32). Then, using the lawn culture method, the bacterial suspension was inoculated onto Muller-Hinton agar (MHA, Oxoid, Ltd., UK). The following antibiotics disks were used as Amoxicillin/clavulanic acid (20/10 μg), Cefotaxime (30 μg), Ceftazidime (30 μg), Ceftriaxone (30 μg), and Cefoxitin (30 μg), Gentamicin (10 μg), Meropenem (10 μg), Tetracycline (30 μg), Ciprofloxacin (30 μg), Chloramphenicol (30 μg), and Trimethoprim/Sulphamethoxazole (5 μg) (BD USA). After that, the plates were incubated for 24 h at 37°C. Following overnight incubation, the zone of inhibition was measured and classified according to CLSI, 2020 as sensitive, intermediate, or resistant (33). The isolates’ multi-drug resistance patterns were categorized using the standards established by Magiorakos et al. (34).

### Quality control

Before data collection, the questionnaire was pretested and the data collectors were given training to guarantee the accuracy of the data. The collected data was reviewed daily for consistency and accuracy. Participants were given instructions on how to appropriately collect samples. Additionally, we checked the culture media, antibiotic discs, and other reagents’ expiration dates ahead of time. By inoculating well-known strains *E. coli* American type culture collection (ATCC) 25922, *S. typhimurium* ATCC 14028 (35), and *S. flexnerii* ATCC 12022 (36), the culture media’s quality was evaluated. Every laboratory procedure was carried out per standard operating protocols (37).

### Statistical analysis

At first, all data were coded and reviewed for accuracy. After coding, the data were entered using EPI-info version 7. For further analysis, the data was transferred to SPSS version 26. For categorical variables, frequency distributions and percentages were calculated. The socio-demographic factors and the prevalence of *Salmonella*, *Shigella*, or intestinal parasites among the study participants were analyzed using Bivariable logistic regression. To determine factors that are statistically significantly associated with the presence of *Salmonella*, *Shigella*, or intestinal parasite infections, variables with *p* ≤ 0.2 in the Bivariable logistic regression analysis have proceeded to the multivariable logistic regression analysis. A *p*-value of <0.05 was considered statistically significant at a 95% confidence level.

### Ethical consideration

Ethical clearance was obtained from the Ethical Review Committee of the School of Biomedical and Laboratory Sciences, College of Medicine and Health Sciences, University of Gondar. A support letter was obtained from the Department of Medical Microbiology at the University of Gondar with reference number SBLS/02/25/2021. Written informed consent was obtained from each study participant. Strict confidentiality was maintained during the interview process and anonymity was kept during data processing and report writing. For the proper parasitic and antimicrobial treatments, food handlers who tested positive for enteric infections (bacterial and parasite) were linked healthcare facility at the University of Gondar.

## Results

### Socio-demographic characteristics of the study participants

In this study, 290 food handlers participated with a 100% response rate. The average age of the study participants was 32.1 years (standard deviation: ±8.7 years), with the majority of them 240 (82.8%) falling between the ages of 20 and 40. The majority 242 (76.9%) of these were females. Regarding educational status, 135 (46.6%) completed secondary school and above. Of the total respondents, 116 (40%), do not have a practice of hand washing after the toilet. 181 (62.4%) of the total respondents had medical checks. Approximately over 71 (24.5%) of the participants in the study had a practice of consuming unpasteurized milk, while 59 (20.3%) and 148 (51%) regularly consumed raw meat and vegetables, respectively (Table 1).

**Table 1:** Socio-demographic characteristics of the study participants at the University of Gondar Cafeterias, Northwest Ethiopia, 2021.

Variables	Category	Frequency n (%)
Gender	Male	48 (16.6)
	Female	242 (83.4)
Age in years	<20	8 (2.8)
	20–40	240 (82.8)
	>40	42 (14.4)
Education level	Illiterate	57 (19.6)
	Primary	98 (33.8)
	Secondary and above	135 (46.6)
Marital status	Unmarried	131 (45.2)
	Married	159 (54.8)
Service years	1–5	262 (90.3)
	Greater than 5	28 (9.7)
Medical checkups	Yes	181 (62.4)
	No	109 (37.6)
Hand washing habit after toilet	Yes	213 (73.5)
	No	77 (26.5)
Hands wash with soap	Yes	227 (78.3)
	No	63 (21.7)
Fingernail status	Trimmed	273 (94.1)
	Not trimmed	17 (5.9)
Wear hair garment	Yes	259 (89.3)
	No	31 (10.7)
practice of consuming unpasteurized milk	Yes	71 (24.5)
	No	219 (75.5)
Eating raw meat	Yes	59 (20.3)
	No	231 (79.7)
Eating raw vegetables	Yes	148 (51)
	No	142 (49)

### The magnitude of *Salmonella*, and *Shigella* species, and intestinal parasites in stool samples from food handlers

**Figure 2:**
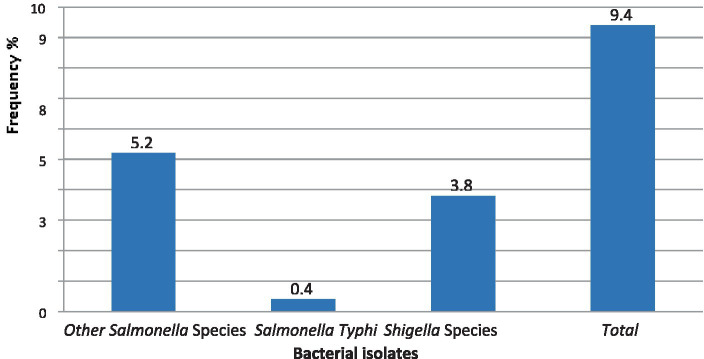
The magnitude of *Salmonella* and *Shigella* species in stool samples from food handlers at the University of Gondar cafeterias, Northwest Ethiopia, 2021.

**Figure 3:**
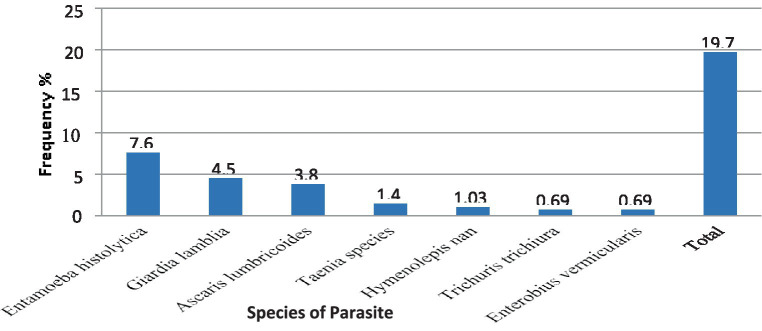
The magnitude of intestinal Parasites in stool samples from food handlers at the University of Gondar Cafeterias, Northwest Ethiopia, 2021.

The overall combined magnitude of *Salmonella* and *Shigella* isolates was 27 (9.3%). The magnitude of *Salmonella* and *Shigella* species in this study was 16 (5.5%) and 11 (3.8%), respectively (Figure 2). In this study, there was at least one intestinal parasite in 290 of the samples analyzed 57 (19.65%). The most detected intestinal Parasitosis was *E. histolytica/dispar* 22 (7.6%), followed by *G. lamblia* 13 (4.5%) *Ascaris lumbricoides* 11 (3.8%), and the least detected intestine parasitosis was 2 (0.69%) *Trichuris trichiura* and *Enterobius vermicularis*. The mixed infections of *E. histolytica/dispar* and *G. lamblia* were observed in 7% of the samples (Figure 3).

### Associated risk factors of *Salmonella* and *Shigella*, and intestinal parasite infection

After bivariate logistic regression analysis, the following factors were added to a multivariate logistic regression analysis: the practice of washing hands after using a toilet (*p* ≤ 0.001), the practice of consumption of unpasteurized milk (*p* ≤ 0.000), and consumption of raw vegetables (*p* ≤ 0.012). The practice of not washing hands after using the toilet (AOR: 4.42, 95% CI: 1.57, 10.56), and the practice of consuming unpasteurized milk (AOR: 3.14, 95% CI: 1.65, 3.96), were factors significantly associated with the prevalence of *Salmonella*, and *Shigella* infection (Table 2). After bivariate logistic regression analysis, the following factors were added to a multivariate logistic regression analysis: the practice of washing hands after using a toilet (*p* ≤ 0.007), the practice of consumption of unpasteurized milk (*p* ≤ 0.000), and consumption of raw vegetables (*p* ≤ 0.015). The practice of not washing hands after using the toilet (AOR: 2.19, 95% CI: 1.0, 1.4), and the practice of consuming unpasteurized milk (AOR: 10.4, 95% CI: 3.8, 28.8), were factors significantly associated with the prevalence of *intestinal parasites* infection (Table 3).

**Table 2:** Associated risk factors of *Salmonella* and *Shigella* infection among the study participants at the University of Gondar Cafeterias, Northwest Ethiopia, 2021.

Variables	Frequency (n, %) *N* = 290	Presence of *Salmonella* and *Shigella* species, (*N* = 290)		COR (95%CI)	*p*-value	AOR (95%CI)	*p*-value
		Presence n (%)	Absence n (%)				
Gender							
Male	48 (16.6)	10 (3.5)	38 (13)	1			
Female	242 (83.4)	17 (5.9)	225 (77. 6)	1.48 (0.67–3.30)	0.329		
Age in years							
<20	8 (2.8)	4 (1.4)	4 (1.4)	1			
20–40	240 (82.8)	9 (3.1)	231 (79.7)	1.84 (0.57–5.94)	0.303	0.50 (0.21–1.20)	0.123
>40	42 (14.4)	14 (4.8)	28 (9.7)	0.62 (0.34–1.12)	0.118	0.98 (0.17–5.38)	0.983
Education level							
Illiterate	57 (19.6)	13 (4.5)	44 (15.1)	0.38 (0.04–3.72)	0.411		
Primary	98 (33.8)	8 (2.8)	90 (31)	1.07 (0.38–2.97)	0.892		
Secondary and above	135 (46.6)	6 (2)	129 (44.4)	1			
Marital status							
Unmarried	131 (45.2)	12 (4.2)	119 (41)	1			
Married	159 (54.8)	15 (5.2)	125 (50)	1.5 (0.16–13.96)	0.704		
Service years							
1–5	262 (90.3)	19 (6. 6)	231 (83.8)	0.84 (0.47–1.51)	0.572		
Greater than 5	28 (9.7)	8 (2.8)	20 (6.8)	1			
Medical checkups							
Yes	181 (62.4)	10 (3.4)	171 (59)	1			
No	109 (37.6)	17 (5.9)	92 (31.7)	0.84 (0.42–1.66)	0.624		
Hand washing practice after toilet							
Yes	213 (73.5)	11 (3.8)	202 (69.7)	1		1	
No	77 (26.5)	16 (5.5)	61 (21)	5.17 (3.01–10.49)	<0.001	4.42 (1.57–10.56)	0.001*
Hands wash with soap							
Yes	227 (78.3)	7 (2.4)	220 (75.9)	1			
No	63 (21.7)	20 (6.9)	43 (14.8)	0.87 (0.49–1.54)	0.644		
Fingernail status							
Trimmed	273 (94.1)	14 (4.8)	259 (89.3)	1		1	
Not trimmed	17 (5.9)	13 (4.5)	4 (1.4)	1.59 (0.49–5.13)	0.433	0.69 (0.2–2.5)	0.566
Wear hair garment							
Yes	259 (89.3)	20 (6.8)	239 (82.5)	1		1	
No	31 (10.7)	7 (2.4)	24 (8.3)	0.06 (0.34–1.95)	0.993	0.86 (0.3–2.7)	0.794
Practice of consuming unpasteurized milk							
Yes	71 (24.5)	49 (16.9)	22 (7.6)	4.29 (1.85–4.05)	0.000	3.14 (1.65–3.96)	0.001*
No	219 (75.5)	8 (2.8)	211 (72.8)	1		1	
Eating raw meat							
Yes	59 (20.3)	8 (2.8)	51 (17.6)	1.58 (0.77–3.23)	0.211	0.60 (0.24–15)	0.282
No	231 (79.7)	19 (6.6)	212 (73)	1		1	
Eating raw vegetables							
Yes	148 (51)	20 (6.9)	128 (44.1)	1.21 (0.09–16.05)	0.012	0.4 (0.2–1.26)	0.723
No	142 (49)	7 (2.4)	135 (46.6)	1		1	

**Table 3:** Associated risk factors of intestinal parasite infection among the study participants at the University of Gondar Cafeterias, Northwest Ethiopia, 2021.

Variables	Frequency (n, %), *N* = 290	Presence of intestinal parasites (*N* = 290)		COR (95%CI)	*p*-value	AOR (95%CI)	*p*-value
		Presence n (%)	Absence n (%)				
Gender							
Male	48 (16.6)	21 (7.1)	27 (9.4)	1		1	
Female	242 (83.4)	36 (12.5)	206 (71)	2.46 (0.84–7.23)	0.100	0.3 (0.08–1.2)	0.086
Age in years							
<20	8 (2.8)	3 (1.0)	5 (1.7)	1			
20–40	240 (82.8)	49 (16.9)	176 (58.6)	0.60 (0.10–3.61)	0.577		
>40	42 (14.4)	15 (5.2)	42 (14.6)	1.03 (0.43–2.48)	0.954		
Education level							
Illiterate	57 (19.6)	22 (7.6)	35 (12.1)	0.16 (0.31–0.97)	0.020		
Primary	98 (33.8)	11 (3.8)	87 (30)	1.70 (0.71–1.92)	0.371		
Secondary and above	135 (46.6)	24 (8.3)	111 (38.3)	1			
Marital status							
Unmarried	131 (45.2)	23 (7.9)	108 (37.2)	1			
Married	159 (54.8)	34 (11.7)	125 (43.1)	0.84 (0.44–1.60)	0.595		
Service years							
1–5	262 (90.3)	31 (10.7)	231 (79.7)	0.28 (0.38–0.95)	0.398		
Greater than 5	28 (9.7)	26 (8.9)	2 (0.7)	1			
Medical checkups							
Yes	181 (62.4)	10 (3.5)	171 (59)	1			
No	109 (37.6)	47 (16.2)	62 (21.4)	0.52 (0.26–1.0)	0.075		
Hand washing practice after toilet							
Yes	213 (73.5)	41 (14.1)	172 (59.4)	1		1	
No	77 (26.5)	16 (5.5)	61 (21)	3.02 (1.36–6.71)	0.007	2.19 (1.03–1.4)	0.001*
Hand washing soap							
Yes	227 (78.3)	27 (9.3)	200 (69)	1			
No	63 (21.7)	30 (10.3)	33 (11.4)	0.84 (0.44–1.60)	0.595		
Fingernail status							
Trimmed	273 (94.1)	49 (16.9)	224 (77.2)	1		1	
Not trimmed	17 (5.9)	8 (2.8)	9 (3.1)	1.59 (0.49–5.13)	0.433	0.69 (0.2–2.5)	0.566
Wear hair cover							
Yes	259 (89.3)	38 (13.1)	221 (76.2)	1		1	
No	31 (10.7)	19 (6.6)	12 (4.1)	1.0 (0.36–2.78)	0.993	0.86 (0.3–2.7)	0.795
Practice of consuming unpasteurized milk							
Yes	71 (24.5)	49 (16.9)	22 (7.6)	8.9 (4.48–17.71)	0.000	10.4 (3.8–28.8)	0.001*
No	219 (75.5)	8 (2.8)	211 (72.8)	1		1	
Eating raw meat							
Yes	59 (20.3)	26 (8.9)	33 (13.3)	1.58 (0.77–3.23)	0.211	0.60 (0.24–15)	0.282
No	231 (79.7)	31 (10.7)	200 (69)	1		1	
Eating raw vegetables							
Yes	148 (51)	47 (16.2)	101 (34.8)	0.44 (0.22–0.85)	0.015	0.4 (0.2–1.26)	
No	142 (49)	10 (3.5)	132 (45.5)	1		1	

*p less than 0.05; COR, crude odds ratio; AOR, adjusted odds ratio; CI, confidence interval; N, the number of study participant.

### Antimicrobial resistance profiles of Salmonella and Shigella isolates

The following antibiotics were tested against *Salmonella* and *Shigella* isolates Amoxicillin/clavulanic acid, Cefotaxime, Ceftazidime, Ceftriaxone, and Cefoxitin, Gentamicin, Meropenem, and were used to assess the susceptibility profile of 16 *Salmonella* and 11 *Shigella* isolates and the results revealed that all isolates were susceptible to Meropenem 11 (100%). Of the Shigella isolates, 11 (100%) to tetracycline and Trimethoprim/Sulphamethoxazole were resistant, followed by 10 (91%) to Amoxicillin/clavulanic acid and 9 (82%) to gentamycin. Furthermore, the *Salmonella* isolates were resistant to tetracycline; Trimethoprim/Sulphamethoxazole and Amoxicillin/clavulanic acid were 7 (46.7%) (Table 4).

**Table 4:** Antimicrobial resistance profile of Salmonella and Shigella isolates among study participants at the University of Gondar Cafeterias, Northwest Ethiopia, 2021.

Bacterial isolates (*N* = 27)	Patterns	Antimicrobials										
		AMC	CHL	CIP	TET	CAZ	CTX	CTR	GEN	SXT	MER	CXT
		N (%)	N (%)	N (%)	N (%)	N (%)	N (%)	N (%)	N (%)	N (%)	N (%)	N (%)
*Salmonella Typhi* (*N* = 1)	S	0	1 (100)	0	0	1 (100)	1 (100)	1 (100)	0	0	1 (100)	1 (100)
	I	0	0	0	0	0	0	0	0	0	0	0
	R	1 (100)	0	1 (100)	1 (100)	0	0	0	1 (100)	1 (100)	0	0
*Shigella species* (*N* = 11)	S	1 (9)	5 (46)	8 (73)	0	9 (82)	10 (91)	9 (82)	2 (18)	0	19 (100)	19 (100)
	I	0	0	0	0	0	0	0	0	0	0	0
	R	10 (91)	6 (54)	3 (27)	11 (100)	2 (18)	1 (9)	2 (18)	9 (82)	11 (100)	0	0
*Salmonella species* (*N* = 15)	S	7 (46.7)	13 (86.7)	14 (93.3)	8 (53.3)	10 (67)	11 (73.3)	8 (53.3)	10 (67)	8 (53.3)	15 (100)	13 (86.7)
	I	1 (6.6)	0	0	0	0	0	0	0	0	0	0
	R	7 (46.7)	2 (13.3)	1 (6.7)	7 (46.7)	5 (33)	4 (26.7)	7 (46.7)	5 (33)	7 (46.7)	0	2 (13.3)
Total (*N* = 27)	S	8 (29.6)	19 (70.4)	22 (81.5)	8 (29.6)	20 (74.1)	22 (81.5)	18 (67)	12 (44.4)	8 (29.6)	37 (100)	25 (92.6)
	I	1 (3.7)	0	0	0	0	0	0	0	0	0	0
	R	18 (66.7)	8 (29.6)	5 (18.5)	19 (70.4)	7 (25.9)	5 (18.5)	9 (33)	15 (55.6)	19 (70.4)	0	2 (7.4)

S, susceptibility; I, intermediate; R, resistance; AMC, Amoxicillin/clavulanic acid; CHL, Chloramphenicol; CIP, Ciprofloxacin; TET, Tetracycline; CAZ, Ceftazidime; CTX, Cefotaxime; CTR, Ceftriaxone; GEN, Gentamicin; SXT, Trimethoprim/Sulphamethoxazole; MER, Meropenem; CXT, Cefoxitin.

In this study, drug resistance to at least three different classes of antimicrobial agents is referred to as multidrug resistance, the overall multidrug resistance was 16 (59.3%), and out of the 27 isolates, 8 (53.3%) *Salmonella* and 7 (63.6%) *Shigella* species were multidrug-resistant isolates (Table 5).

**Table 5:** The magnitude of Multidrug-resistant *Salmonella* and *Shigella* isolates among study participants at the University of Gondar Cafeterias, Northwest Ethiopia, 2021.

Antimicrobial classes		Bacterial isolates			
		*Shigella* species	*Salmonella Typhi*	*Salmonella* species	Total (*N* = 27)
R0 (All drug sensitive)		1	–	3	4
R1 (for One)		0	0	0	0
R2 (For Two)	AMC, SXT (Not MDR)	3	–	–	3
	AMC, GEN (Not MDR)	0		1	1
	AMC, TET (Not MDR)	0		3	3
R3 (For Three)	AMC, GEN, CHL (MDR)	2			2
	CTR, SXT, TET (MDR)	0		1	1
	AMC, TET, GEN (MDR)	1		3	4
	AMC, GEN, SXT (MDR)	1		1	2
	AMC, SXT, TET, CIP (MDR)	1		1	2
	AMC, SXT, TET, CRO (MDR)	1	1	1	3
R5 (For Five)	AMC, SXT, TET, CHL, CTR (MDR)	0	0	1	1
R6 (For Six)	AMC, TET, SXT, CHL, CTR, CAZ (MDR)	1			1
	Total Non-MDR isolates N (%)	4 (36.4)	0	7 (46.7)	11 (40.7)
	Total MDR-isolates N (%)	7 (63.6)	1 (100)	8 (53.3)	16 (59.3)

CHL, Chloramphenicol; CIP, Ciprofloxacin; TET, Tetracycline; CAZ, Ceftazidime; CTX, Cefotaxime; CTR, Ceftriaxone; GEN, Gentamicin; SXT, Trimethoprim/Sulphamethoxazole; AMC, Amoxicillin/clavulanic acid; MDR, bacterial isolates resistant to three or more antibiotics classes.

## Discussion

Poor food handling practices can cause food contamination, which in turn may result in food-borne diseases that could represent a risk to the health of the general population. Food handlers play a major role in the spread of pathogens (38–40).

In our study, 27 (9.3%) of *Salmonella* or *Shigella* species were detected in food handlers. This finding is similar to those of previous studies performed in Ethiopia in which the reported isolation rate was 5.9% in Debre Markos University, Ethiopia (41) and 10% in Arba Minch, Ethiopia (42). But, our result is lower than those reported from Nigeria (62.6%) (43), and Sudan (30.1%) (44), and higher than that reported from Ethiopia in Gondar 3.1% (45) and 3.5% in Addis Ababa (46), and in Japan (0.032%) (47). The discrepancies may be due to differences in sample sizes, research participant characteristics, and pathogen isolation techniques. This incidence of enteric pathogens in the current investigation suggests that the food handlers’ cleanliness standards were put to the test because enteric pathogens like *Shigella* and *Salmonella* species were detected from stool cultures (41). The cornerstone of limiting the spread of infections from food handlers to consumers is appropriate hygiene habits, and knowledge status, both individually and when handling food (42, 45, 48).

In this study, the magnitudes of *Salmonella* species among food handlers were 16 (5.5%). This was comparable to studies carried out in Northern Ethiopia, Adigrat (7.3%) (39), Southern, Ethiopia, Arba Minch (6.9%) (42), and Nigeria, (5.5%) (49). However, our result was higher than the studies reported from Ethiopia, Addis Ababa (3.5%) (46), Bahir Dar (1.6%) (23), and Gondar (3.1%) (50). However, our results were lower than those of research conducted in Ethiopia, Dilla (9.5%) (21), Addis Ababa (10.5%) (22), and Nigeria (42.3%) (43). The discrepancy could be explained by the research sites’ varying levels of environmental sanitation and inadequate sanitary conditions.

In this study, the isolation rate of *Shigella* species was 3.8%; it can be a sign of inadequate food handler cleanliness and result in bacillary dysentery epidemics among the population of students. The results of the research are supported by the findings in Ethiopia, Arba Minch (3%) (42), Dilla (3.2%) (21), and Gondar (2.7–3.1%) (45, 51). Nevertheless, less than the results of other research done in Nigeria (15.5%) (43). These might result from the food handlers’ irregular training in food preparation, handling, and cleanliness procedures, as well as variations in study years.

In this study, the frequency of parasite infection was found to be 19.65%. This agrees with studies conducted in Ghana (21.6%) (10); however, it is higher than what was found in research carried out in Sudan (6.9%) (44) and Iran (11.9%) (52). Nevertheless, it is lower compared to the findings in Ethiopia, Bahir Dar (41.1%) (23) and Dilla (38.6%) (21), in Anatolia (52.2%) (53), in Nigeria (38.1%,) (43), and India (29.3%) (54). The discrepancy could be explained by variations in study years, sociodemographic characteristics, Study area, sanitary conditions, environmental sanitation, safe water supply, health promotion practices, food hygiene and safety training, and awareness of intestinal parasite transmission and prevention.

In this investigation, *E. histolytica/dispar* was the main parasite found in 7.6% of the stool samples analyzed. This is consistent with earlier research that was done in Ethiopia, where this parasite was found most frequently (15, 55). *E. histolytica* is one of the typical protozoan parasites that cause human gastroenteritis, along with *Giardia lamblia* 4.5% (56). It spreads mostly by fecal-oral contact, and in areas where poor hygiene standards are prevalent, contaminated hands play a significant part in its transmission (57, 58).

In this study, Food handlers who did not have a hand-washing practice after the toilet were two times more likely to be positive for intestinal parasites and *Shigella* and *Salmonella* species compared to those who had a hand-washing habit after using the toilet. Food handlers who drank unpasteurized milk were 10 times more likely to be positive for intestinal parasites and Shigella and *Salmonella* species compared to those who drank pasteurized milk. Food handlers this means that most of the intestinal parasitosis, Salmonellosis, and Shigellosis were transmitted by lack of hand washing practice after toilet and drunken unpasteurized milk. This study is a consistent study with Ethiopia, Gondar (51), Bahir Dar (59), Motta (60), Dilla (21), and Mekelle (61). Poor hygienic environments, inadequate toilets, and a lack of readily available facilities for hand washing practices all contribute to the spread of *Salmonella* and *Shigella* species. The majority of the university’s food handlers reported only washing their hands with water, and some of them admitted to skipping the step entirely after using the bathroom (62).

In this study, regarding antimicrobial susceptibility test results, a high rate of isolate susceptibility was observed to a few of the antimicrobial agents which is comparable to the study conducted at Debre Markos University, Ethiopia (41). However, *Shigella* and *Salmonella* isolates showed a high resistance rate to tetracycline, Trimethoprim/Sulphamethoxazole, and Augmentin which is concordant with the previous report from Gondar Town Health Institution, Ethiopia (63). This may have been caused by the extensive and unnecessary use of antibiotics. In the current practice of Ethiopia, it is simple for the general people to walk into a pharmacy and purchase antibiotics off the market. The high resistance rates seen could be explained by this indiscriminate usage of antibiotics (64).

In this study, *Shigella* species were highly resistant to Tetracycline and Trimethoprim/Sulphamethoxazole (100%), Chloramphenicol (54%), and Amoxicillin/clavulanic acid (91%). This is similar to the study conducted in Ethiopia, Haramaya, that reported resistance of *Shigella* species to Tetracycline (76.2%), Chloramphenicol (66.7%) (25), and in Tigrai that reported resistance of Shigella species to Tetracycline (50%), Amoxicillin/clavulanic acid (100%), and Chloramphenicol (50%) (58). Additionally, it was comparable research from Arbaminch, Ethiopia found that the Shigella species were 40% of Amoxicillin/clavulanic acid (65). Another study conducted at the Gondar University cafeteria also showed that 75% of tetracycline and chloramphenicol, followed by Trimethoprim/Sulphamethoxazole (50%) and Gentamicin (25%) (66) and at catering establishments at Debre Markos University the result showed that 62.5% of Tetracycline, 25% Chloramphenicol, and (37.5%) Trimethoprim/Sulphamethoxazole (41).

The *Salmonella* species were also resistant to many of the antibiotics in the present study: 46.7% to Tetracycline, Amoxicillin/clavulanic acid, and Trimethoprim/Sulphamethoxazole. This is comparable with a study done in Ethiopia, Gondar (51), Debre Markos University (41), Tigrai (58), and Dilla (21), which indicated that antimicrobial resistance of Salmonella species is an increasing concern. These results suggested that Ethiopian antimicrobial resistance is still a problem that has to be addressed. Antibiotic resistance has emerged as a significant global public health problem, necessitating coordinated action (67). Antibiotic resistance is mostly caused by changes in bacterial genomes, inappropriate antibiotic use, poor drug regulation rules, incorrect drug prescriptions, and disobedience to prescriptions (68).

In this study, *Salmonella* isolates were highly susceptible to Ceftriaxone which was comparable with previous reports in Ethiopia (21, 69). The magnitude of MDR Shigella and Salmonella species in this study was 63.6 and 53.3%, respectively, which is comparable with the previous findings in Ethiopia (21, 69) but lower than 100% resistance in Addis Ababa, Ethiopia (46). The high MDR rate of *Salmonella* and *Shigella* isolates for most of the antibiotics currently used could limit our antibiotic options for empirical therapy.

### Limitations

Due to a lack of available materials, the *Shigella* and *Salmonella* isolates in our investigation could only be identified at the species level. Furthermore, due to financial constraints, it was not possible to differentiate between the *Entamoeba* complex species (*E. dispar* and *E. histolytica*) by using molecular methods.

## Conclusion and recommendations

In this study, the overall prevalence of *Salmonella* species and *Shigella* species was high as compared to the previous reports. *Salmonell*a species was highly resistant to Trimethoprim/Sulphamethoxazole, amoxicillin-clavulanic acid, and Tetracycline, however highly susceptible to Meropenem, Ciprofloxacin, Cefoxitine, and Chloramphenicol. *Shigella* species also show a high resistance rate to Trimethoprim/Sulphamethoxazole, Tetracycline, amoxicillin-clavulanic acid, and Gentamicin, and showed the least resistance to Ceftazidime, and Cefotaxime. Among the total Salmonella and Shigella isolates, multidrug resistance was documented in 59.3% of all bacterial isolates. The overall prevalence of different intestinal parasites from stool samples was 19.65%. Among those, the most prevalent intestinal parasitosis was *E. histolytica/dispar*, followed by *G. lamblia*. Regarding risk factors, consumption of unpasteurized milk, and no hand washing practice after the toilet showed high significant correlation with intestinal parasites, *Shigella*, or *salmonella* infection. Through continuing health education and training programs, the level of knowledge, as well as the standard of conduct of food handlers at cafeteria facilities, must be improved. To improve the personal hygiene of the food handlers, it is also advised that the required facilities be provided, such as a clean toilet and a safe water supply. Additionally, to prevent microbial infections in food handlers, it is recommended that consumers drink pasteurized milk, and constant epidemiological surveillance is recommended.

## Data availability statement

The raw data supporting the conclusions of this article will be made available by the authors, without undue reservation.

## Ethics statement

The studies involving humans were approved by Ethical Review Committee of the School of Biomedical and Laboratory Sciences, College of Medicine and Health Sciences, University of Gondar. The studies were conducted in accordance with the local legislation and institutional requirements. The participants provided their written informed consent to participate in this study.

## Author contributions

AzA: Conceptualization, Data curation, Formal analysis, Investigation, Methodology, Project administration, Software, Visualization, Writing – original draft, Writing – review & editing. SE: Methodology, Supervision, Validation, Writing – review & editing. DK: Investigation, Software, Supervision, Validation, Visualization, Writing – review & editing. AyA: Data curation, Investigation, Writing – original draft. WA: Investigation, Supervision, Validation, Writing – original draft, Writing – review & editing. FM: Conceptualization, Software, Supervision, Validation, Visualization, Writing – review & editing.
